# Efficacy, safety and tolerability of Lisdexamfetamine Dimesylate Treatment Compared to Placebo in Adults with Binge-Eating Disorder: A Systematic Review and Meta-Analysis of Randomized Controlled Trials

**DOI:** 10.1192/j.eurpsy.2025.1387

**Published:** 2025-08-26

**Authors:** M. Prätzel Ellwanger, V. Astori, B. Westphalen Pomianoski, D. Lopes Vieira, M. Frizzo Messinger

**Affiliations:** 1 Universidade do Contestado, Mafra; 2 Escola Superior de Ciências da Santa Casa de Misericórdia de Vitória, Vitória; 3 Universidade Nove de Julho, São Paulo; 4 Universidade Federal de Minas Gerais, Belo Horizonte; 5 Universidade Federal do Rio Grande do Sul, Porto Alegre, Brazil

## Abstract

**Introduction:**

Binge-Eating Disorder (BED) is characterized by frequent episodes of consuming excessive amounts of food, leading to both psychological and physical symptoms. Treatment typically involves a combination of psychotherapy and antidepressants. The disorder is often associated with dysfunctions in the dopamine and norepinephrine systems and to address these dysfunctions, lisdexamfetamine dimesylate (LDX) may offer potential benefits by targeting impulse control and reward pathways, thereby addressing these underlying issues.

**Objectives:**

This study aims to evaluate the efficacy, safety, and tolerability of LDX compared to placebo in adults with BED through a systematic review and meta-analysis.

**Methods:**

We systematically searched PubMed, Embase, and Cochrane Central for randomized controlled trials (RCTs) comparing LDX versus placebo in patients with BED. Primary outcome was binge eating days per week (BEDW) and secondary outcomes were Yale–Brown Obsessive–Compulsive Scale modified for binge eating (YBOCS-BE), Clinical global impressions-improvement scale (CGI-I), weight reduction (WR) and specific occurrence of treatment-emergent adverse event (TEAEs), like dry-mouth and insomnia. Mean differences (MDs), standardized mean differences (SMDs) and risk ratio (RR) were used for all outcomes. p<0.05 presented significant statistical results, while I²>40% represented a high heterogeneity.

**Results:**

A total of 5 RCTs were included, involving a total of 963 patients, of whom 517 patients received LDX. BEDW (MD: -1.29; 95% CI [-1.65, -0.93]; p<0.01; I²=60%; Figure 1A) was significantly reduced when comparing LDX with placebo. YBOCS-BE (MD: -6.16; 95% CI [-8.35, -3.97]; p<0.01; I²=66%; Figure 1B) has shown an indication of reduction of obsessive-compulsive behaviors (OCB) in patients using LDX. CGI-I (RR: 1.72; 95% CI [1.12, 2.63]; p=0.032; I²=71%; Figure 2A), WR (SMD: -1.31; 95% CI [-1.55, -1.07]; p<0.01; I²=59%; Figure 2B). The use of LDX exhibit an increase on dry-mouth (RR: 5.08; 95% CI [3.39, 7.61]; p=0.001; I²=0%; Figure 3A) and insomnia (RR: 3.00; 95% CI [1.52, 5.94]; p=0.014; I²=0%; Figure 3B) when compared with placebo.

**Image 1:**

**Figure:**
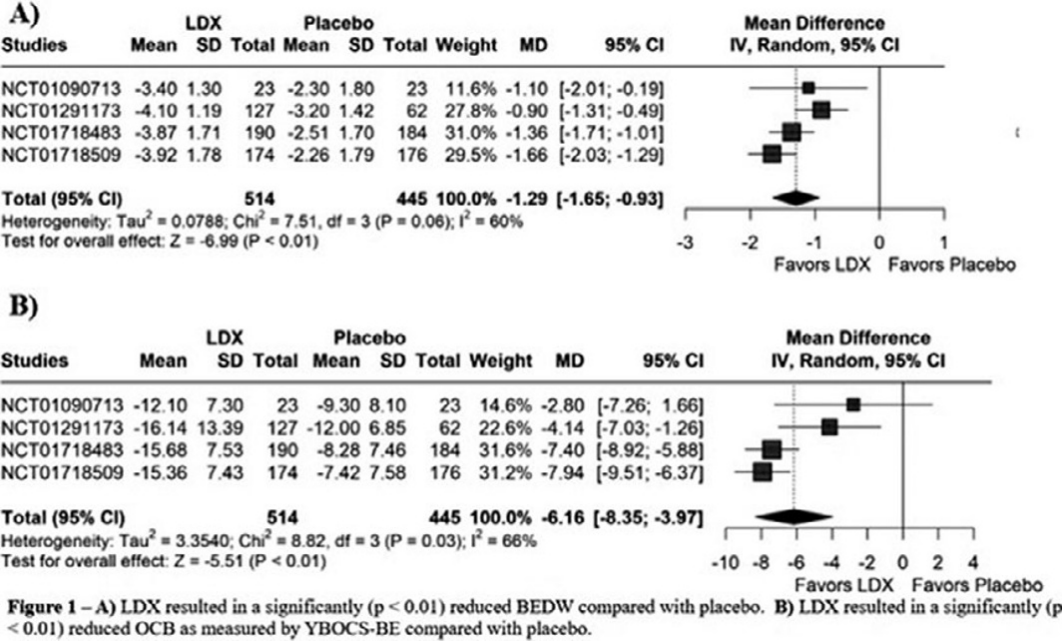

**Image 2:**

**Figure:**
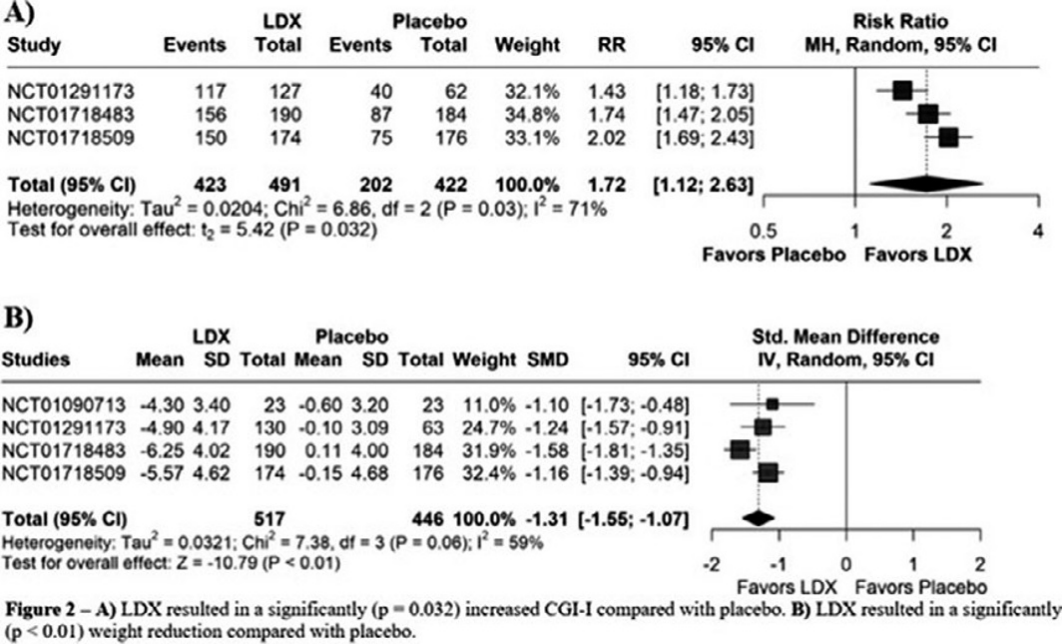

**Image 3:**

**Figure:**
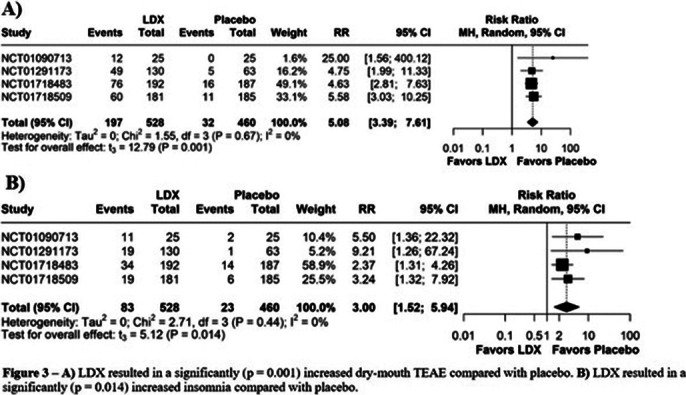

**Conclusions:**

Our study has shown significant improvements in the use of LDX in BED. Patients presented a reduction in OCB, CGI-I, WR, and BEDW. Although some TEAEs were observed, LDX treatment in BED shows a greater benefit.

**Disclosure of Interest:**

None Declared

